# The Emerging Role of Gβ Subunits in Human Genetic Diseases

**DOI:** 10.3390/cells8121567

**Published:** 2019-12-04

**Authors:** Natascia Malerba, Pasquelena De Nittis, Giuseppe Merla

**Affiliations:** 1 Division of Medical Genetics Unit, IRCCS Casa Sollievo della Sofferenza, Viale Cappuccini, 71013 San Giovanni Rotondo (FG), Italy; n.malerba@operapadrepio.it; 2 Center for Integrative Genomics, University of Lausanne, CH-1015 Lausanne, Switzerland; pasquelena.denittis@unil.ch

**Keywords:** heterotrimeric G-proteins, β subunits, neurodevelopmental disorders, human genetic diseases

## Abstract

Environmental stimuli are perceived and transduced inside the cell through the activation of signaling pathways. One common type of cell signaling transduction network is initiated by G-proteins. G-proteins are activated by G-protein-coupled receptors (GPCRs) and transmit signals from hormones, neurotransmitters, and other signaling factors, thus controlling a number of biological processes that include synaptic transmission, visual photoreception, hormone and growth factors release, regulation of cell contraction and migration, as well as cell growth and differentiation. G-proteins mainly act as heterotrimeric complexes, composed of alpha, beta, and gamma subunits. In the last few years, whole exome sequencing and biochemical studies have shown causality of disease-causing variants in genes encoding G-proteins and human genetic diseases. This review focuses on the G-protein β subunits and their emerging role in the etiology of genetically inherited rare diseases in humans.

## 1. G-Protein-Coupled Receptors (GPCRs) and Heterotrimeric G-Proteins

The G-Protein-Coupled Receptor (GPCR) superfamily includes over 800 members in humans [1] and is the largest group of cell-surface seven-transmembrane receptors [2]. They translate the signal from extracellular ligands into intracellular responses [3]. The GPCRs have a ligand-binding pocket, with seven motif α-helices, in the extracellular region, and a cytoplasmic domain engaged in G-proteins binding, guanosine triphosphate (GTP)-binding heterotrimers, consisting of α, β, and γ subunits [4,5].

**Figure 1:**
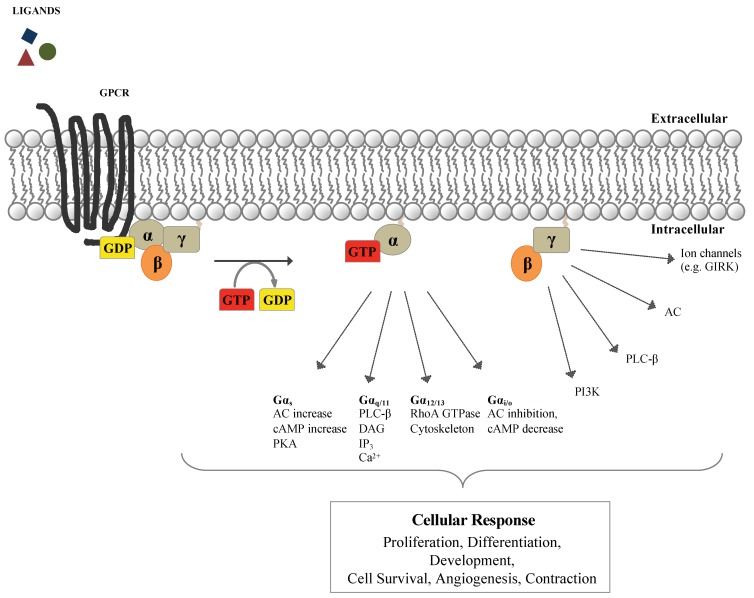
G-protein-coupled receptors signalosome. In the resting state, G-proteins are heterotrimers of alpha bound to guanosine diphosphate (GDP, yellow), beta, and gamma subunits. When activated by an extracellular ligand through G-protein coupled receptors (GPCRs, black), they undergo a conformational change that permits the GDP exchange with GTP (red) on Gα, which then dissociates from Gβγ. In the active state, Gα-GTP and Gβγ regulate various effectors. According to functional and structural homologies of their α subunit, heterotrimeric G-proteins are divided into four types (Gα_s_, Gα_i/o_, Gα_q/11_, and Gα_12/13_). Each Gα defines the unique Gαβγ mediated cellular responses [1,13,14,15,16]. Gα_s_ and Gα_i_ subfamily members are involved in the modulation of the intracellular second-messenger cAMP levels, either stimulating (G_s_) or inhibiting (G_i_) the production of cAMP by AC activity. Gα_q/11_ induces the activation of PLC-β, promoting the production of the intracellular messenger DAG and IP_3_ which activate the PKC and calcium signaling. Gα_12/13_ plays a role in the activation of the RhoA GTPase and of phospholipase D in regulating cell shape and motility [14,17,18]. Adenylyl cyclase (AC); cyclic adenosine monophosphate (cAMP); protein kinase A (PKA); phospholipase Cβ (PLC-β); diacylglycerol (DAG); inositol (1,4,5) trisphosphate (IP_3_); protein kinase C (PKC); intracellular concentration of free Ca^2+^ (Ca^2+^); Ras homolog family member A GTPase (RhoA GTPase); phosphatidylinositol-3-kinase (PI3K); G-protein–gated inwardly rectifying potassium channels (GIRK).

When inactive, the G-protein α subunit is linked to guanosine diphosphate (GDP). Ligand-activated GPCRs catalyze the exchange of GDP with GTP on Gα, promoting its dissociation from Gβγ (Figure 1). The Gαβγ dissociation, in turn, promotes the activation of the Gα and Gβγ units that activate downstream factors, thus regulating an array of cellular functions such as cell contraction, excitability, migration, cell growth, and differentiation [6,7]. Notably, the combinatorial association of the distinct G-protein subunit subtypes, comprising at least 20 Gα, 5 Gβ, and 13 Gγ subunits [8,9], provides the level of selectivity that is needed to generate the wide range of signals governed by G-proteins and their cognate GPCRs (Figure 1) [10,11,12].

The propagation of the GPCR signaling cascade is restricted by the Regulators of G-protein Signaling (RGS) proteins, which limit the active Gα subunit lifetime and accelerate its GTP hydrolysis with a consequent re-association with the Gβγ dimer [19,20,21,22,23,24].

Here, we review the Gβ subunits and their contribution to the etiology of rare human genetic conditions. In the last six years, the outbreak of Next Generation Sequencing (NGS) technologies has assisted us to reach the description of a tapestry of human genetic conditions caused by the pathogenic variants in Gβ subunits, and disease manifestations mainly involving neuronal and cardiac systems associated with ophthalmic pathology.

## 2. Gβ Subunits: Genes and Proteins Structure

The human genome contains five genes (*GNB1* to *GNB5*) encoding the different Gβ subunits [25]. Chromosomal locations, genes structure and exons content of each of the five subunits are summarized in Table 1. The Gβ_1-4_ subunits share between 80 and 90% sequence identity and are widely expressed throughout the tissues [26,27]; the Gβ_5_ exhibits much less homology (~50%) and is preferentially expressed in the brain and nervous system [28], while the Gβ_5_ longer isoform, Gβ_5_L, has restricted expression in retinal photoreceptor outer segments [9,29].

At the protein level, iconic is the beta-propeller structure of the Gβ subunits, characterized by seven regular WD40-repeats [30] and a coiled coil domain at the N-terminus end. The WD40 domain is one of the most abundant and interacting domain in the eukaryotic proteome; each domain is approximately 40 amino acids long and is characterized by a conserved tryptophan (W)-aspartic (D) acid pair, hence the name WD40 [29,31]. With its β-propeller architecture, the WD40 domain provides extensive surface exposure for protein-protein or protein-DNA interaction, that coordinate downstream cellular events including signal transduction, autophagy, and apoptosis [32].

## 3. Gβ proteins and Human Diseases

### 3.1. G Protein Subunit Beta 1 (GNB1, Gβ_1_)

In humans, heterozygous *GNB1* (MIM 139380) missense, splice-site and frameshift pathogenic variants cause an autosomal dominant neurodevelopmental disorder, named MRD42 (Mental Retardation, Autosomal Dominant 42; MIM#616973). The phenotype observed across individuals with MRD42 include global developmental delay (GDD)/intellectual disability (ID), hypotonia often associated with limb hypertonia, various types of seizures, and poor overall growth [35,36,37]. Strabismus, nystagmus, cortical visual impairment, attention deficit hyperactivity disorder, and autistic features may also be present [38]. Less frequent and variable symptoms are ataxia, dystonia, hydronephrosis, acute lymphoblastic leukemia [35,37,38,39,40], and cutaneous mastocytosis [41,42].

*GNB1* was found as one of the five genes deleted in five patients with 200 to 823-kb overlapping interstitial deletions of chromosome 1p36.33 (MIM#607872) affected by ID, developmental delay, seizures and muscular hypotonia together with characteristic dysmorphic features, and behavior abnormalities [43,44]. Functional evidence of *GNB1* involvement in neurodevelopmental delay is also corroborated by the study of homozygous *Gnb1* mutant mice, demonstrating that *Gnb1* is essential for normal embryonic neurogenesis. Forty percent of *Gnb1* knock-out embryos were neonatal lethal and showed defects in neural tube closure and neural progenitor cell proliferation associated to exencephaly (Table 2); embryos without neural tube defects presented microencephaly and died after birth [45]. Moreover, *Gnb1* heterozygous mice exhibited abnormal retina morphology with progressive degeneration (http://www.informatics.jax.org/marker/MGI:95781), thus supporting the ophthalmic manifestations reported in MRD42 affected individuals.

**Figure 2:**
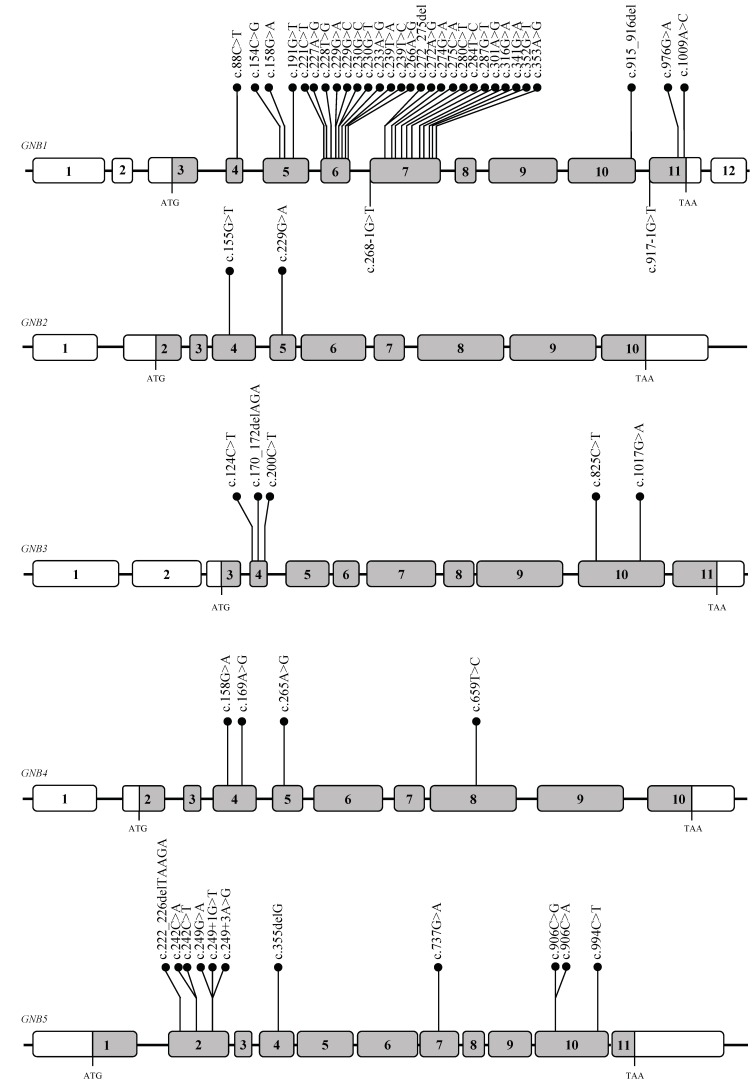
Variants distribution across the entire *GNB1-GNB5* genes. Genomic coordinates are specified on the GRCh37.p13 genome assembly. Coding exons are indicated by grey boxes, while untranslated regions are displayed in white. Variants annotations refer to NM_002074 for *GNB1*, NM_005273 for *GNB2*, NM_002075 for *GNB3*, NM_021629 for *GNB4*, NM_006578 for *GNB5*.

To date, twenty eight *de novo* and four with undefined inheritance *GNB1* variants have been reported in 53 affected individuals; of these 28 are missense, 2 frameshift, and 2 splice-site variants (Figure 2) [35,36,37,38,39,40,41,42].

Overall, 24/32 (~75%) *GNB1* variants affect residues coded by exon 6 (11/24) or exon 7 (13/24) (Figure 2). This small part of the gene encodes for a protein region forming the Gα and Gβγ interaction surface [46]. Accordingly, three *GNB1* likely benign missense variants (c.88C>T, p.(Leu30Phe); c.272A>G, p.(His91Arg); c.1009A>C, p.(Lys337Gln) are located distantly from the interaction site and no impaired Gβ_1_ functionality has been shown [40].

### 3.2. G Protein Subunit Beta 2 (GNB2, Gβ_2_)

Heterozygous *GNB2* (MIM 139390) c.155G>T, p.(Arg52Leu) (Figure 2) missense variant has been identified in 11 individuals of a family composed of 25 members. Carriers were affected by an autosomal dominant form of Sinus Node Dysfunction (SND) occurring in combination with atrioventricular conduction dysfunction and atrial fibrillation, in the absence of heart structural problems [47]. Crystal structure model of the mammalian G-protein–coupled inwardly rectifying potassium channel 2 (GIRK2) with β_1_γ_2_ G-protein complex, showed that Arg52 lays at the binding interface with GIRK [48], a data confirmed also for GIRK1/4 and Gβ_2_ [47]. Therefore, the presence of the mutant residue is predicted to decrease the steric interaction at the GIRK-Gβ_2_ surface. Functional studies revealed that the heterozygous variant has an impact on the rectification of the GIRK channel with a consequent increase of ACh-activated K^+^ current (I_K,ACh_) [47], thus displaying a gain-of-function effect. Of note, the cardiac GIRK channels are directly switched on by the Gβγ units and are involved in the negative chronotropic effect of the parasympathetic nervous system, thus controlling heart rate and cellular electrical excitability [49,50]. A recent study of 52 unrelated patients with idiopathic SND uncovered a nonsynonymous substitution (c.303G>C, p.(Trp101Cys)) in the *KCNJ5* gene, encoding the Kir3.4 subunit of the GIRK channel. The mutation leads to a sustained activation of the cardiac GIRK channel [51]. Finally, further examples of the connection between *GNB2* and heart disease are provided by the *Gnb2* knock-out mice, generated by the International Mouse Phenotyping Consortium (IMPC; https://www.mousephenotype.org/data/genes/MGI:95784) [52]. Null *Gnb2* mice showed an increased heart rate, and abnormal electrocardiogram line, revealing shortened RR interval, PQ interval, and ST segment (Table 2).

Additionally, an individual with global developmental delay, intellectual disability, muscle hypotonia and dysmorphisms carrying a *de novo GNB2* missense variant (c.229G>A, p.(Gly77Arg)) (Figure 2), predicted to impair protein function, was recently described in [53]. This study highlights that *GNB2* variants not only associate to cardiac manifestations, but cause developmental delay too [53].

### 3.3. G Protein Subunit Beta 3 (GNB3, Gβ_3_)

Homozygous and compound heterozygous *GNB3* (MIM 139130) disease-causing variants were described in three individuals of a large Lebanese–Armenian family affected by Congenital Stationary Night Blindness type 1H (CSNB1H, MIM#617024) [8] and in a fourth sporadic case [69]. CSNB refers to a group of clinically heterogeneous retinal disorders caused by genetic defects of the retinoid metabolism in the retinal pigment epithelium (RPE), phototransduction, or signal transmission through the bipolar cells (BCs) [70,71]. Based on BCs ability to either initiate or terminate light stimuli, BCs can be either ON- or OFF-type. Specifically, while cone photoreceptors can connect both ON- and OFF-BCs, the rods are served largely by the ON-BCs [70].

The three first identified *GNB3* variants lie in the first (c.170_172delAGA, p.(Lys57del); c.200C>T, p.(Ser67Phe)) and seventh (c.1017G>A, p.(Trp339*)) WD40 repeat of the encoded Gβ_3_ protein, respectively (Figure 2). Homology model studies of Gβ_3_ protein structure, pointed out that each variant would impact interactions abilities as well as the formation of effective G-protein complexes [8]. A fourth *GNB3* variant (c.124C>T, p.(Arg42Ter)) was found in a patient with distinct early-onset inherited retinal disease, characterized by nystagmus, normal funduscopic exam, full-field electroretinography abnormalities, and mild disturbance of the central macula [69]. The Arg42 variant, located in exon 4 of the gene, gives rise to a premature stop codon, which is expected to be a substrate of the nonsense–mediate decay pathway [69].

Gβ_3_ is expressed at higher levels in the eyes, in particular in the cone photoreceptors and ON-BCs of the retina in mammals and additional species [72,73,74]. In the eye, Gβ_3_ modulates cone transducing function and cone and rod ON-bipolar signaling [8].

Similar to humans [8], abnormal light ON bipolar response and reduced cone sensitivity was also found in a *Gnb3* knock-out mouse model [75,76], while retinopathy globe enlarged *(rge)* phenotype was reported in the chicken carrying a 3-bp homozygous deletion of the *GNB3* homolog [77]. Interestingly, ablation of the *Gnb3* gene in mice causes mild bradycardia [58], thus suggesting a possible additional role of *GNB3* in heart rate regulation.

Beyond the role of *GNB3* pathogenic variants in the etiology of CSNB1H, [8] Siffert and colleagues [78], described the c.825C>T (rs5443) polymorphism in exon 10 of the gene as linked to the expression of a shortened splice variant, Gβ_3_s, whose translated protein is characterized by the deletion of 41 amino acids, responsible of enhanced G-proteins signal transduction [78]. The c.825C>T polymorphism is associated with an increased risk of hypertension [78,79,80,81], obesity [59,82], diabetes [83], metabolic syndrome component [84,85], depression [86,87], seasonal variations in mood and behaviors [88], functional dyspepsia [89,90], stroke [91,92], arrhythmia [93], coronary artery disease [94,95], and other cardiovascular phenotypes [96,97,98,99]. In addition, duplication and overexpression of *GNB3* gene is responsible for a syndromic form of childhood obesity [59,100].

### 3.4. G Protein Subunit Beta (GNB4, Gβ_4_)

Heterozygous pathogenic variants in *GNB4* gene (MIM 610863) (Figure 2) have been reported as causative of intermediate Charcot–Marie–Tooth disease F (CMTDIF, MIM#615185), an autosomal dominant form of CMT. CMT is a neurologic disorder characterized by progressive distal muscle atrophy and weakness and variable nerve conduction velocities ranging from the demyelinating to the axonal range [101]. Heterozygous c.158G>A, p.(Gly53Asp) *GNB4* missense variant was reported in six affected family members. An unrelated case carried the heterozygous c.265A>G; p.(Lys89Glu) *de novo* missense variant [101]. The pathogenicity of the variants and the importance of GPCR signaling in peripheral-nerve function in humans were supported by the reduced Gβ_4_ immunostaining in the axon and Schwann cells of peripheral nerves of affected individuals. Moreover, in vitro studies demonstrated that both variants altered the bradykinin induced GPCR signaling [101].

More recently, the description of one Czech patient presenting the c.169A>G, p.(Lys57Glu) variant [102], and one Japanese family, for which axonal neuropathy has been reported, and segregating with c.659T>C, p.(Gln220Arg) [103], confirmed the pathogenic role of *GNB4* as causal gene of CMTIDF.

All the *GNB4* pathogenic variants described so far are located in the first (p.(Gly53Asp) and p.(Lys57Glu)) [101,102], in the second (p.(Lys89Glu)) [101], and in the fifth WD40 domain (p.(Gln220Arg)) [103], respectively (Figure 2). The Gly53 and Lys89 are important residues for the architecture of the WD40 β-propeller structure [104]. Functional characterization of p.(Gly53Asp) and p.(Lys89Glu) showed an impaired GPCR signaling via a dominant-negative effect, and resulting in reduced PLCβ_2_ activity [104,105] followed by inhibition of IP_3_ production and moderate increase in cytosolic calcium (Ca^2+^) level [101,106], a universal second messenger that regulates the transmission of the depolarizing signal and neuronal synaptic activity.

Similar to Gβ_2_, Gβ_4_ is known to influence the activity of the cardiac GIRK channel, which regulates the heart rhythm through the acetylcholine-dependent activation of the muscarinic M2-receptor present in the sinoatrial node [107,108,109,110]. Although this gene has been reported in human hereditary neuropathy, genome-wide association studies have revealed association of the *GNB4* locus with variation in heart rate [47,111,112]. This suggests that *GNB4* variation may also impact heart rate.

### 3.5. G Protein Subunit Beta (GNB5, Gβ_5_)

The *GNB5* gene (MIM 604447), encoding the subunit β5 of the heterotrimeric G-proteins, is a divergent member of the Gβ family with distinct biochemical properties. Differently from Gβ_1–4_, Gβ_5_ forms irreversible dimer with the G-protein γ-like (GGL) domain [113] present in the R7 regulator group of G-protein signaling proteins (R7 RGS) [64,114,115,116,117,118,119]. Interaction of the GGL domain and the atypical Gβ_5_ is a general requirement for stabilization of the whole R7 protein subfamily.

Homozygous or compound heterozygous variants in the *GNB*5 gene have been associated with either IDDCA (Intellectual Developmental Delay with Cardiac Arrhythmia, MIM#617173) or LADCI (Language delay and ADHD/Cognitive Impairment with or without cardiac arrhythmia, MIM#617182) human syndromes [66,120,121,122,123,124,125]. Homozygous carriers of the recurrent missense variant c.242C>T, p.(Ser81Leu), present with LADCI syndrome, characterized by mild intellectual disability in combination with language delay, attention-deficit/hyperactivity disorder, with or without cardiac arrhythmia [66,125]. The substitution of the evolutionary conserved Serine 81 with the hydrophobic Leucine was predicted to compromise protein folding and/or stability as well as impair the binding kinetics of RGS proteins [66] and their capacity to deactivate G-protein signaling initiated by dopamine receptors [125]. By contrast, homozygous or compound heterozygous carriers of *GNB5* Loss of Function alleles presented IDDCA, whose phenotypic spectrum includes epileptic seizures, severe intellectual disability, drastic impairment in speech and language skills, vision problems (which mainly include nystagmus and retinal abnormalities), hypotonia, and sick sinus syndrome [66,120,121,122,123,124]. Among the *GNB5* pathogenic variants described so far [66,120,121,122,123,124,125], a mutational hot spot in exon 2, encoding the first WD40 domain and containing 58% of described variants, has been identified (Figure 2). The evidence of the *GNB5* involvement in neuronal and cardiac signaling was confirmed in *Gnb5*-null zebrafish and mouse models that resulted in neuronal and cardiac phenotypes reminiscent of those of IDDCA patients [63,66,126,127].

*Gnb5*-null mouse models displayed marked neurobehavioral abnormalities, impaired gait and motor learning, hyperactivity [62,63,64,65], defective visual adaptation with perturbed development and functioning of retinal bipolar cells [127,128,129]. Moreover, targeted deletion of one or two copies of the *Gnb5* gene had distinct effects on body weight and behavior in mice [62]. Although the cardiac phenotype of *Gnb5*-null mouse has never been studied, it is interesting to observe that bradycardia and heart rate responses to the cholinergic stimulation were exhibited by mice lacking *Rgs6*, the *Gnb5*-dependent RGS protein in the heart [130,131,132]. The *gnb5* knock-out zebrafish model also recapitulated the phenotypic spectrum of affected individuals, highlighting the involvement of *GNB5* in the control of motor capacity, vision and heart rate [66]. Several model organisms have been characterized regarding *GNB5*; information of additional animal models is included in Table 2.

## 4. Concluding Remarks

Heterotrimeric G-protein signaling is one of the most important mechanisms of cellular communication. They are involved in a vast array of cellular processes required for the normal growth and development of cells. The Gβ proteins, representing one of the components of the heterotrimeric G-proteins, are specifically expressed in different tissues and elicit a wide range of specialized cellular responses. It is not surprising that mutations altering the G-proteins function, compromise cellular responses and associate with aberrant physiological functions, resulting in disease.

We anticipate that unravelling the role of Gβ proteins in neurodevelopmental and cardiac conditions may help to provide targeted strategies to effectively modulate their pathogenesis and to shed light on possible future therapeutic approach.

## Figures and Tables

**Table 1:** Gene content and major features of the five genes encoding the Gβ subunits. Gene names are reported according to the Hugo Gene Nomenclature Committee (HGNC, [33]); Ensembl gene and transcript IDs, information on transcript/protein length as well as number of exons were retrieved to the Ensembl 97 and Ensembl Genomes 44 release, and, finally, genomic coordinates are specified on the GRCh38.p13 genome assembly. Uniprot identifiers rely on the UniProt release 2019_06 (published July 3, 2019) [34]. MIM IDs and phenotype MIM numbers are as in OMIM (Online Mendelian Inheritance in Men) database.

Gene Name (HGNC)	Description	Ensembl ID	RefSeq ID	Ensembl Transcript ID	Transcritp Length (bp)	Protein length (aa)	Uniprot	Cytogenetic Location	Genomic Coordinates (GRCh38, from Ensembl)	Strand	Nr. of Exons	Nr. of Coding Exons	MIM ID	Phenotype MIM Number(s)
*GNB1*	G protein subunit beta 1	ENSG00000078369.18	NM_002074	ENST00000378609.9	3163	340	P62873	1p36.33	1:1,785,285-1,891,117	reverse strand	12	9	*139380	#616973
*GNB2*	G protein subunit beta 2	ENSG00000172354.10	NM_005273	ENST00000303210.9	1664	340	Q6FHM2	7q22.1	7:100,673,567-100,679,174	forward strand	10	9	*139390	-
*GNB3*	G protein subunit beta 3	ENSG00000111664.10	NM_002075	ENST00000229264.7	1923	340	P16520	12p13.31	12:6,840,211-6,847,393	forward strand	11	9	*139130	#617024
*GNB4*	G protein subunit beta 4	ENSG00000114450.10	NM_021629	ENST00000232564.8	6315	340	Q9HAV0	3q26.33	3:179,396,088-179,451,476	reverse strand	10	9	*610863	#615185
*GNB5*	G protein subunit beta 5	ENSG00000069966.18	NM_006578	ENST00000358784.11	1735	353	O14775	15q21.2	15:52,122,206-52,180,001	reverse strand	11	11	*604447	#617173, #617182

**Table 2:** GNB genes have been studied in different model organisms. The table lists phenotypic manifestations resulting from complete (knock-out, KO) or partial (knock-down, KD) lack of each of the five GNB genes. “HET” refers to mouse models carrying only one functional copy of the gene, and “Dup” concerns the presence of three copies. Of note, in Zebrafish each of the genes has two paralogs, as a result of an ancient genome duplication event. In *Drosophila melanogaster* and *C. elegans* only two definite homologues have been identified, one corresponding to human *GNB1-4* and one corresponding to human *GNB5*, in each species. NA indicates “Not Available” model.

Gene Name (HGNC)	Annotated Terms in Animal Models			
	*M. Musculus*	*Zebrafish*	*D. Melanogaster*	*C. elegans*
*GNB1*	(***Gnb1***): abnormal brain morphology and size (KO) [52]	(***gnb1a/gnb1b***): altered regulation of neutrophil migrations and posterior lateral line neuromast primordium migration (KD) [54]	(***CG10545***): abnormal spindle size (KD and overexpression) [55]	(***gpb1***): essential for embryo development (50–80% embryonic lethality); uncoordinated phenotype in surviving adult worms; functions in establishment of mitotic spindle orientation; expressed in alimentary system, body wall musculature, epithelial system, nervous system and reproductive organs (KD) [56,57]
*GNB2*	(***Gnb2***): abnormal behavioral response to light, increased heart rate, shortened PQ interval, shortened RR interval, shortened ST segment (KO) [52]	NA		
*GNB3*	(***Gnb3***): abnormal eye electrophysiology, mild bradycardia (KO); weight gain (Dup) [58,59,60]	(***gnb3a***): expressed throughout development; (***gnb3b***) expressed in the cones of the dorsal and medial retina (KD) [61]		
*GNB4*	(***Gnb4***): enlarged heart and spleen (KO) [52]	NA		
*GNB5*	(***Gnb5***): pre-weaning lethality with incomplete penetrance, decreased body size, slow post-natal weigh gain and abnormal vision (KO); increased body weight, adiposity, insulin resistance and liver steatosis (HET) [62,63,64,65]	(***gnb5a/gnb5b***): abnormal heart contraction, optokinetic behavior and swimming behavior (KO) [66]	(***CG10763***): pain responsive defective (KD) [67]	(***gpb-2***): behavioral defects, e.g., delayed egg laying, locomotion, and pharingeal pumping (KO and overexpression) [68]
